# Retention rate of TNF inhibitors versus IL-17 inhibitors in ankylosing spondylitis patients with prior biologics experience

**DOI:** 10.1186/s13075-025-03601-z

**Published:** 2025-07-04

**Authors:** Jiwon Yang, Bong-Woo Lee, Youngjae Park, Ji Hyeon Ju, Wan-Uk Kim, Sung-Hwan Park, Seung-Ki Kwok, Jennifer Jooha Lee

**Affiliations:** Division of Rheumatology, Department of Internal Medicine, Seoul St. Mary’s Hospital, College of Medicine, The Catholic University of Korea, 222 Banpo-daero, Seocho-gu, Seoul, 06591 Republic of Korea

**Keywords:** Ankylosing spondylitis, TNF inhibitors, IL-17 inhibitors, Biologics, Second or higher line, Drug retention, Real-world data

## Abstract

**Background:**

For patients with ankylosing spondylitis (AS) who experience inefficacy or adverse events with biologics, no recommendations exist regarding the preference for class cycling or switching as a second- or higher-line biologics. Previous studies on the drug retention of TNF and IL-17 inhibitors in AS patients with prior biologics exposure have limitations, including relatively short follow-up periods, exclusion of patients with extra-articular manifestations, and a primary focus on second-line treatment. This study aimed to compare the retention rates of TNF and IL-17 inhibitors in AS patients with prior biologics experience, over a relatively longer follow-up period in real clinical practice.

**Methods:**

A total of 148 AS cases receiving either a TNF or IL-17 inhibitor as a second- or higher-line biologic were retrospectively analyzed after propensity score matching. Patient characteristics at the time of cycling or switching and drug retention were compared between the two groups. Subgroup analyses were conducted based on the reasons for drug discontinuation. Cox regression analyses were used to identify the factors associated with drug discontinuation.

**Results:**

The median follow-up period was 31.4 months, and drug survival tended to be lower for IL-17 inhibitors than for TNF inhibitors in the Kaplan–Meier analysis (*P* = 0.134). The lower retention of IL-17 inhibitors was more pronounced when discontinuations unrelated to treatment failure were censored (*P* = 0.034) or when used for reasons other than psoriasis aggravation (*P* = 0.028). However, in multivariable Cox regression, the number of previous biologics (HR: 1.62, 95% CI: 1.17–2.23, *P* = 0.003) and BASDAI (HR: 1.3, 95% CI: 1.03–1.65, *P* = 0.030) were significantly associated with drug discontinuation, whereas the class of biologics did not reach statistical significance (HR: 2.11, 95% CI: 0.96–4.63, *P* = 0.064).

**Conclusion:**

In patients with AS who had prior experience with biologics, the drug retention of TNF inhibitors tended to be higher compared to IL-17 inhibitors in real-world clinical practice. However, the factors significantly associated with higher drug survival were a lower number of previously exposed biologics and a lower BASDAI.

**Supplementary Information:**

The online version contains supplementary material available at 10.1186/s13075-025-03601-z.

## Introduction

Ankylosing spondylitis (AS) is a chronic inflammatory disorder primarily involving the axial skeleton and often accompanied by peripheral arthritis or extra-articular manifestations such as uveitis, psoriasis, and inflammatory bowel disease (IBD) [1, 2]. Non-steroidal anti-inflammatory drugs (NSAIDs) are effective in alleviating symptoms and serve as the initial treatment for AS [1]. For patients with persistently high disease activity despite conventional treatment, the most recent 2022 recommendations by the Assessment of SpondyloArthritis International Society (ASAS) and the European Alliance of Associations for Rheumatology (EULAR) suggest considering tumor necrosis factor (TNF) inhibitors, interleukin 17 (IL-17) inhibitors, or Janus kinase (JAK) inhibitors [3]. In current practice, TNF or IL-17 inhibitors are generally preferred to JAK inhibitors as first-line biologics due to extensive evidence and clinical experience in their use [3].

Although no clear priority is established between TNF or IL-17 inhibitors, it is recommended that a monoclonal antibody against TNF should be preferred in patients with a history of recurrent uveitis or active IBD, whereas an IL-17 inhibitor may be preferred in patients with significant psoriasis [3]. However, there is no currently recommended guideline for the decision of second- or higher-line biologics. While switching to a different class is conditionally recommended if a biologic or targeted synthetic disease-modifying anti-rheumatic drug fails in rheumatoid arthritis [4, 5], related research in AS remains limited and inconsistent.

Studies have shown that TNF and IL-17 inhibitors exhibit comparable drug retention and clinical efficacy over a 1-year follow-up period in AS patients with prior exposure to biologics [6–8]. A similar study of five Nordic rheumatology registries also indicated that drug retention and clinical efficacy were comparable between secukinumab and adalimumab as first- or second-line treatments; however, secukinumab showed inferior performance as a third- or higher-line treatment [9]. Min et al. reported that secukinumab had lower drug retention than alternative TNF inhibitors in secondary non-responders to prior TNF inhibitors [10]. In studies with longer follow-up periods, Kwon et al. reported that alternative TNF inhibitors and secukinumab did not show a significant difference in drug survival as second-line treatments over a median observation period of 27.8 months. However, this study excluded patients with extra-articular manifestations due to their influence on treatment choice [11]. Recently, Van Es et al. suggested that the 3-year drug retention rate was higher in the TNF inhibitor cycling group than in the IL-17 inhibitor swapping group after the failure of the initial TNF inhibitor [12]. This study included patients who underwent cycling or swapping between 2012 and 2023. Thus, bias may have occurred because of the local prescribing practices in the Netherlands, where a cycling strategy was predominantly used before 2019 and a swapping strategy afterward.

Previous studies on the drug retention of TNF and IL-17 inhibitors in AS patients with prior biologics exposure have limitations, including relatively short follow-up periods, exclusion of patients with extra-articular manifestations, and a primary focus on second-line treatment.

In this study, we aimed to compare the retention rates of TNF and IL-17 inhibitors as second- or higher-line treatments in AS patients with prior biologics experience over a relatively extended follow-up period in real-world clinical practice. To minimize time bias, we included patients with comparable timing of biologic cycling or switching.

## Methods

### Study population and data collection

All data used in the present study were obtained from the medical records of patients with AS who visited Seoul St. Mary’s Hospital, a university-affiliated tertiary referral center in Korea. The diagnosis of AS was based on the modified New York criteria [13]. Considering that IL-17 inhibitors were first introduced at our center in March 2019, patients with AS who underwent TNF inhibitor or IL-17 inhibitor cycling or switching from March 2019 onward were included. To ensure a minimum follow-up period of six months, the class cycling or switching point was restricted to January 2024, with data collected up to August 2024 for analysis. The treatment for all patients, including the selection and duration of biologic therapies, was determined at the discretion of each of the seven treating physicians. Each cycling or switching event was regarded as a distinct case. Consequently, a single patient who underwent multiple biologic changes during the study period could contribute multiple cases. This study was approved by the Institutional Review Board of Seoul St. Mary’s Hospital, Catholic University of Korea (KC24RISI0694).

Data on patient demographics, clinical characteristics, and disease-specific metrics were collected. The key clinical variables required for analysis had been systematically and comprehensively recorded using a standardized electronic case report form (eCRF) system, allowing for a reliable and consistent chart review. These included age, sex, human leukocyte antigen B27 (HLA-B27) positivity, smoking status, body mass index (BMI), disease duration, comorbidities, number of previously exposed biologics, reasons for cycling or switching biologics, disease activity or functional indices, and the presence of peripheral or extramusculoskeletal manifestations. Disease activity and functional indices assessed were the Bath Ankylosing Spondylitis Disease Activity Index (BASDAI) [14], Ankylosing Spondylitis Disease Activity Score (ASDAS) [15], and Bath Ankylosing Spondylitis Functional Index (BASFI) [16]. Peripheral or extramusculoskeletal manifestations included peripheral arthritis, enthesitis, dactylitis, psoriasis, uveitis, and IBD and were identified based on their occurrence from the time of AS diagnosis to the time of biologic cycling or switching.

Discontinuation of a biologic was defined as either a change to another agent or stopping the biologic treatment. Reasons for discontinuation were categorized as follows: (1) inefficacy, (2) adverse events, and (3) other causes, including symptom improvement, planned or confirmed pregnancy, and cost.

### Statistical analysis

The distribution of continuous variables was assessed using the Kolmogorov–Smirnov test. Variables with a normal distribution were expressed as mean ± standard deviation (SD), whereas those without a normal distribution are reported as median and interquartile range (IQR). Categorical variables were expressed as frequencies and percentages. Student’s *t*-test or Mann–Whitney U-test was used to compare continuous variables, while the chi-squared or Fisher’s exact test was used to compare categorical variables between the two groups. Propensity score (PS) matching was used to adjust for potential confounders due to differences in the baseline characteristics and sample sizes between the TNF and IL-17 inhibitor groups. Baseline differences were observed in BMI and BASDAI scores, with a non-significant difference in the number of previous biologics. PS matching was conducted for age, sex, and BMI, which were considered important demographic factors, along with the BASDAI and the number of previous biologics, which were found to differ between the two groups and influence drug retention.

Kaplan–Meier curves and log-rank tests were used to compare drug retention rates. The Cox proportional hazards model was used to calculate the hazard ratio (HR) for drug discontinuation. To account for the non-independence of observations arising from individual patients contributing multiple treatment cases, Cox proportional hazards models were fitted using robust standard errors clustered by patient ID. Co-variables included age, sex, BMI, HLA-B27 positivity, disease duration, peripheral arthritis, number of previous biologics, and BASDAI scores. Univariable and multivariable analyses were performed using the backward selection method to identify factors associated with discontinuation. Statistical analyses were conducted using the R software (Windows 3.3.2; The R Foundation for Statistical Computing, Vienna, Austria) or SPSS software (version 24.0; IBM Corporation, Chicago, IL, USA). Statistical significance was defined as a *P*-value < 0.05.

## Results

### Baseline characteristics of the study population

In total, 151 consecutive cases of patients with AS were initially included, and the number of cases between the two groups was comparable prior to PS matching: 77 in the TNF inhibitor group and 74 in the IL-17 inhibitor group. After PS matching for age, sex, BMI, BASDAI, and the number of previous biologics, each group included 74 cases, yielding a total of 148 cases. The TNF inhibitor group included 66 cases of TNF inhibitor cycling and eight cases of switching from an IL-17 inhibitor to a TNF inhibitor. In contrast, the IL-17 inhibitor group included 71 cases of switching from a TNF inhibitor to an IL-17 inhibitor and three cases of IL-17 inhibitor cycling. In 92.6% (137/148) of cases, the most recently used biologic agent was a TNF inhibitor. The baseline characteristics of the patients after PS matching are presented in Table 1. Apart from a significantly higher prevalence of psoriasis in the IL-17 inhibitor group (5.4% vs. 28.4%; *P* < 0.001), there were no significant differences observed between the two groups. Although the differences between the two groups were not statistically significant, the TNF inhibitor group had more patients with HLA-B27 positivity and higher BMI, whereas the IL-17 inhibitor group tended to have patients with previous use of a higher number of biologics.

**Table 1:** Baseline characteristics of AS patients on TNF or IL-17 inhibitor as a second- or higher-line treatment

Variables	TNF inhibitor		IL-17 inhibitor	*P*-value
	(*n* = 74)		(*n* = 74)	
Age, years	41.6 ± 12.1		41.9 ± 11.9	0.891
Male, n (%)	58 (78.4)		54 (73)	0.565
HLA-B27 positivity, n (%)	64/67 (95.5)		58/69 (84.1)	0.055
Current smoker, n (%)	19/67 (25.7)		16/69 (21.6)	0.626
BMI, kg/m^2^	25.1 [22.9–27.5]		23.9 [22.2–26.4]	0.101
Disease duration, months	153.0 [63.0–201.0]		106.5 [43.0–197.0]	0.344
Comorbidity				
Hypertension	18 (24.3)		15 (20.3)	0.554
Coronary artery disease	3 (4.1)		4 (5.4)	1
Diabetes	7 (9.5)		12 (16.2)	0.219
Dyslipidemia	34 (45.9)		28 (37.8)	0.317
Osteoporosis	11 (14.9)		16 (21.6)	0.287
Chronic kidney disease	0 (0.0)		1 (1.4)	1
Peptic ulcer	4 (5.4)		3 (4.1)	1
Malignancy	2 (2.7)		6 (8.1)	0.275
Number of previous biologics, n (%)				0.086
1	57 (77.0)		50 (67.6)	
2	14 (18.9)		12 (16.2)	
3	3 (4.1)		9 (12.2)	
4	0 (0.0)		3 (4.1)	
Reason for discontinuation of last biologics, n (%)				0.729
Ineffectiveness	47 (63.5)		50 (67.6)	
Others	27 (36.5)		24 (32.4)	
BASDAI	6.8 ± 1.3		6.3 ± 1.3	0.052
ASDAS-CRP	3.4 ± 1.1		3.3 ± 1.0	0.654
BASFI	2.0 [0.9–5.1]		2.5 [0.4–4.7]	0.919
ESR, mm/hr	8.0 [2.0–28.0]		9.0 [ 2.0–20.0]	0.585
CRP, mg/dL	0.3 [0.1–1.6]		0.5 [ 0.1–1.2]	0.835
Peripheral arthritis, ever	23 (31.1)		35 (47.3)	0.064
Enthesitis, ever	19 (25.7)		18 (24.3)	1
Dactylitis, ever	2 ( 2.7)		2 ( 2.7)	1
Psoriasis, ever	4 ( 5.4)		21 (28.4)	< 0.001^**^
Uveitis, ever	28 (37.8)		17 (23.0)	0.074
IBD, ever	6 ( 8.1)		2 ( 2.7)	0.275

^*^P < 0.05, ^**^P < 0.01

Data are presented as mean ± standard deviation or median [interquartile range] or n (%). AS: ankylosing spondylitis, TNF: tumor necrosis factor, IL-17: interleukin 17, HLA-B27: human leukocyte antigen B27, BMI: body mass index, BASDAI: Bath Ankylosing Spondylitis Disease Activity Index, ASDAS: Ankylosing Spondylitis Disease Activity Score, CRP: C-reactive protein, BASFI: Bath Ankylosing Spondylitis Functional Index, ESR: erythrocyte sedimentation rate, IBD: inflammatory bowel disease

### Reasons for discontinuation

The median follow-up period was 31.4 months (IQR: 15.0–47.2). During this time, 26.4% (*n* = 39) discontinued their biologic treatment. In total, 21.6% (*n* = 16) and 31.1% (*n* = 23) of the cases in the TNF inhibitor and IL-17 inhibitor groups, respectively, discontinued their biologics. The reasons for discontinuation are summarized in Table 2. If multiple reasons for discontinuation were identified, each was analyzed individually. The most common reasons for discontinuation of TNF inhibitors were inefficacy (9.5%), other causes (9.5%), and adverse events (6.8%). In contrast, the leading reasons for discontinuing IL-17 inhibitors were inefficacy (18.9%), adverse events (16.2%), and other causes (8.1%).

**Table 2:** Comparison of reasons for drug discontinuation

	Total (*n* = 148)	TNF inhibitor (*n* = 74)	IL-17 inhibitor (*n* = 74)
Discontinuation	39 (26.4%)	16 (21.6%)	23 (31.1%)
Inefficacy	21 (14.2%)	7 (9.5%)	14 (18.9%)
Adverse events	17 (11.5%)	5 (6.8%)	12 (16.2%)
Other causes^†^	13 (8.8%)	7 (9.5%)	6 (8.1%)

^†^ includes symptom improvement, planned or confirmed pregnancy, and costs

TNF: tumor necrosis factor, IL-17: interleukin 17

### Drug retention and associated factors of discontinuation

**Fig. 1:**
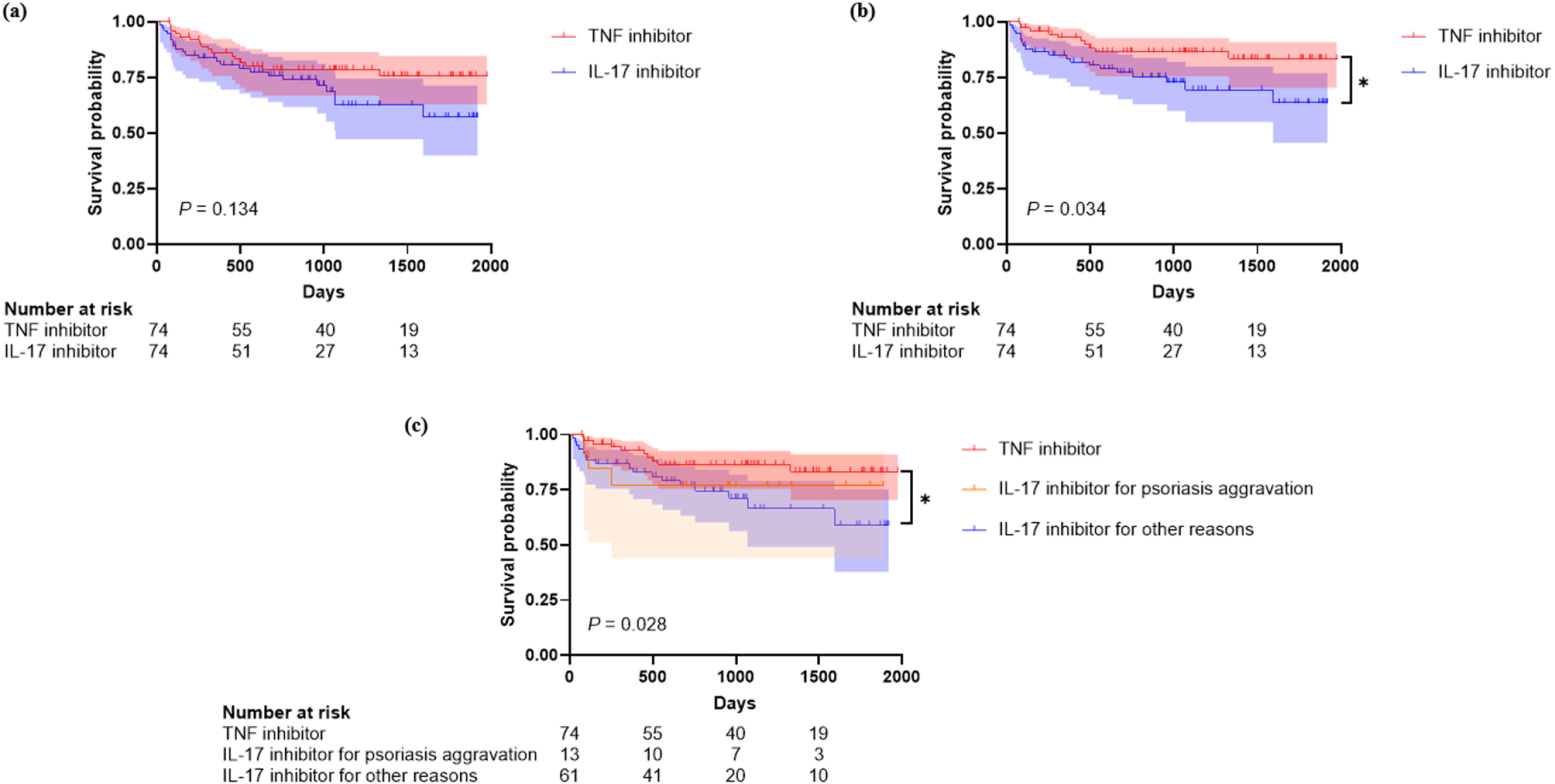
Drug retention of TNF and IL-17 inhibitors as second- or higher-line biologics. (**a**) All cases (PS-matched) (**b**) Censoring discontinuations unrelated to treatment failure (**c**) Subgroup analysis based on reasons for IL-17 inhibitor use. TNF, tumor necrosis factor; IL-17, interleukin 17

The median follow-up duration was 35.7 months (IQR: 16.6–49.9) for the TNF inhibitor group and 28.8 months (IQR: 12.1–38.0) for the IL-17 inhibitor group. Kaplan–Meier curves comparing drug retention of TNF and IL-17 inhibitors in PS-matched cases are shown in Fig. 1a. A total of 141 cases had a follow-up duration of more than one year. Based on Kaplan–Meier analysis, the 1-year drug retention rate was 86.1% (95% confidence interval [CI]: 78.4–94.5) for TNF inhibitors and 82.2% (95% CI: 73.8–91.5) for IL-17 inhibitors. The 2-year retention rates were 78.5% (95% CI: 69.5–88.8) and 75.9% (95% CI: 66.4–86.6), respectively. Among 119 cases with a follow-up duration of more than three years, the 3-year retention rate was significantly higher in the TNF inhibitor group (78.5%, 95% CI: 69.5–88.8) compared to the IL-17 inhibitor group (62.5%, 95% CI: 50.3–77.8). Kaplan–Meier analysis (*P* = 0.134) and the 1- and 2-year retention rates showed no statistically significant differences between the two groups; however, the difference in 3-year retention rates was substantial.

**Fig. 2:**
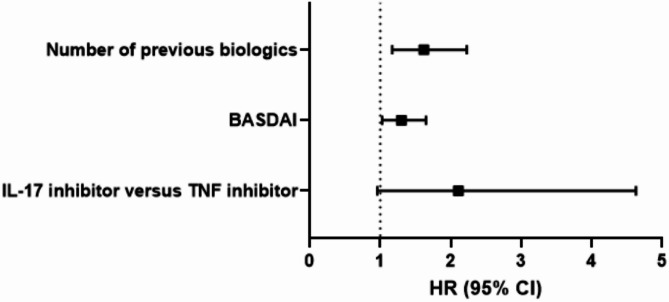
Forest plot of factors associated with discontinuation of biologics. BASDAI, Bath Ankylosing Spondylitis Disease Activity Index; IL-17, interleukin 17; TNF, tumor necrosis factor; HR, hazard ratio; CI, confidence interval

As shown in Table 2, some patients discontinued the drug for reasons unrelated to the drug itself, such as the patient’s will due to symptom improvement. Therefore, we additionally compared the drug retention of TNF and IL-17 inhibitors, censoring discontinuations unrelated to treatment failure, including those due to remission or patient preference. Kaplan–Meier analysis showed that drug survival was significantly higher in the TNF inhibitor group than in the IL-17 inhibitor group (*P* = 0.034, log-rank test) (Fig. 1b). The univariable Cox proportional hazards model identified IL-17 inhibitor use as a significantly associated factor of drug discontinuation (HR: 2.23, 95% CI: 1.06–4.70, *P* = 0.035). Additionally, number of previous biologics (HR: 2.02, 95% CI: 1.49–2.74, *P* < 0.001) and BASDAI (HR: 1.41, 95% CI: 1.09–1.83, *P* = 0.010) were significantly associated with drug discontinuation. Multivariable analysis was performed using the backward selection method, including variables that showed significant associations in univariable analysis (Table 3). The remaining variables were the number of previous biologics, BASDAI, and biologic class. Multivariable analysis revealed the number of previously exposed biologics (HR: 1.62, 95% CI: 1.17–2.23, *P* = 0.003) and BASDAI (HR: 1.30, 95% CI: 1.03–1.65, *P* = 0.030) as factors significantly associated with drug discontinuation. The class of biologics (HR: 2.11, 95% CI: 0.96–4.63, *P* = 0.064) showed only borderline significance. A forest plot summarizing these findings is presented in Fig. 2.

**Table 3:** Factors associated with discontinuation of biologics

Variables	Univariable				Multivariable			
	HR	95% CI		*P*-value	HR	95% CI		*P*-value
Age	1	0.98	1.03	0.741				
Male gender	0.46	0.19	1.08	0.076				
BMI	0.96	0.86	1.06	0.423				
HLA-B27 positivity	1.06	0.94	1.2	0.350				
Disease duration	1	1	1	0.800				
Peripheral arthritis	0.79	0.35	1.8	0.576				
Number of previous biologics	2.02	1.49	2.74	**< 0.001** ^**^	1.62	1.17	2.23	**0.003** ^**^
BASDAI	1.41	1.09	1.83	**0.010** ^*^	1.3	1.03	1.65	**0.030** ^*^
IL-17 inhibitor versus TNF inhibitor	2.23	1.06	4.7	**0.035** ^*^	2.11	0.96	4.63	**0.064**

^*^P < 0.05, ^**^P < 0.01

HR: hazard ratio, CI: confidence interval, BMI: body mass index, HLA-B27: human leukocyte antigen B27, BASDAI: Bath Ankylosing Spondylitis Disease Activity Index, IL-17: interleukin 17, TNF: tumor necrosis factor

### Impact of psoriasis on retention

IL-17 inhibitors have shown superiority in achieving skin outcomes in psoriatic arthritis compared with TNF inhibitors [17, 18], and ASAS-EULAR recommends that IL-17 inhibitors may be preferred in patients with significant psoriasis [3]. Our baseline characteristics reflect this, as the IL-17 inhibitor group had more patients with psoriasis than the TNF inhibitor group (Table 1). To analyze the impact of psoriasis on the retention of IL-17 inhibitors, we conducted a subgroup analysis by dividing the IL-17 inhibitor group into patients who used IL-17 inhibitors for psoriasis aggravation (*n* = 13) and patients who used IL-17 inhibitors for other reasons (*n* = 61). All cases included in the IL-17 inhibitor subgroup for psoriasis aggravation were switchers from TNF inhibitors. The subgroup that used IL-17 inhibitors for other reasons exhibited significantly lower drug retention than that of the TNF inhibitor group (*P* = 0.028). In contrast, the subgroup that used IL-17 inhibitors due to psoriasis aggravation exhibited drug retention comparable to that of the TNF inhibitor group (Fig. 1c). The univariable Cox proportional hazard model identified a significantly increased HR for drug discontinuation in patients who used IL-17 inhibitors for reasons other than psoriasis aggravation (HR: 2.38, 95% CI: 1.08–5.20, *P* = 0.031). However, similar to Table 3, multivariable analysis identified the number of biologics being previously exposed to as the only significant factor associated with drug discontinuation (HR: 1.83, 95% CI: 1.23–2.72, *P* = 0.003) (Supplementary Table S1).

## Discussion

This study showed that TNF inhibitors tended to have a higher drug retention rate compared to IL-17 inhibitors in patients with AS who have prior experience with biologics. This difference in retention appeared to be more pronounced after censoring discontinuations unrelated to treatment failure or among patients who used IL-17 inhibitors for reasons other than psoriasis aggravation. However, in the multivariable analysis adjusting for number of previous biologics and BASDAI, the class of biologics showed borderline statistical significance (*P* = 0.064). The factors significantly associated with TNF or IL-17 inhibitors discontinuation were the number of previously exposed biologics (*P* = 0.003) and BASDAI (*P* = 0.030).

In our study, the median follow-up duration was 31.4 months, with 35.7 and 28.8 months for TNF and IL-17 inhibitors, respectively. Inefficacy was the predominant reason for discontinuation in both groups (9.5% and 18.9%, respectively), consistent with findings from previous studies of patients with AS who experienced TNF inhibitor failure and received TNF or IL-17 inhibitors as second- or higher-line treatments with shorter follow-up periods [6, 8].

At one year, the retention rate was 86.1% (95% CI: 78.4–94.5) for TNF inhibitors and 82.2% (95% CI: 73.8–91.5) for IL-17 inhibitors. Notably, there was no significant difference between the two groups at this time point, consistent with earlier studies reporting comparable 1-year retention rates for TNF- and IL-17 inhibitors in patients with AS previously exposed to TNF inhibitors [6–8]. At three years, the retention rate was 78.5% (95% CI: 69.5–88.8) for TNF inhibitors and 62.5% (95% CI: 50.3–77.8) for IL-17 inhibitors, reflecting significantly lower long-term retention for IL-17 inhibitors. These findings suggest that TNF- and IL-17 inhibitors as second- or higher-line treatments have comparable retention rates in the short-term, but IL-17 inhibitors exhibit significantly lower retention rates in long-term follow-up. Furthermore, when focusing solely on treatment failure, which is defined as discontinuation due to inefficacy or adverse events, IL-17 inhibitors exhibited significantly lower retention rates compared to TNF inhibitors. This finding aligns with a recent study reporting that the 3-year drug retention as second-line biologics following first-line TNF inhibitor failure was significantly higher for TNF inhibitors than for IL-17 inhibitors in patients with AS [12]. Additionally, the drug survival of IL-17 inhibitors used for non-psoriasis-related reasons was significantly poorer than that of TNF inhibitors. In contrast, the drug survival of IL-17 inhibitors used for psoriasis aggravation was comparable to that of TNF inhibitors. IL-17 inhibitors have demonstrated efficacy in psoriatic arthritis, not only in TNF-naïve patients but also in those with inadequate responses or intolerance to TNF inhibitors [19–23]. The therapeutic efficacy of IL-17 inhibitors in psoriatic arthritis, regardless of prior TNF inhibitor exposure, may contribute to the comparability between the drug retention of IL-17 inhibitors used for psoriasis aggravation and that of TNF inhibitors.

Factors such as a higher number of previous biologics and higher BASDAI scores were associated with an increased risk of drug discontinuation, consistent with previous studies [6, 24–27]. The clinical efficacy of TNF or IL-17 inhibitors in AS is known to be lower in patients with a history of biologics exposure than in biologic-naïve patients [28–31]. Linde et al. recently reported that ASDAS-inactive disease rates of third-line TNF inhibitors were lower than those of the second-line [32]. Reduced efficacy of higher-line treatments may have contributed to these outcomes. Since this study was conducted with a relatively small sample size, larger prospective studies focusing on the significance of previous biologics and BASDAI as factors associated with drug discontinuation would be valuable.

This study has some limitations, including its relatively small sample size and retrospective design. To address these limitations and minimize bias, we included consecutive cases during a study period in which the use of both biologics was comparable, and conducted PS-matching and multivariable analyses. Although these methods cannot entirely eliminate limitations, the findings remain clinically relevant as they reflect real-world practices. While robust, randomized controlled trials (RCTs) may lack generalizability due to their controlled environments. In real-world clinical settings, where treatment decisions are influenced by health insurance policies and current management recommendations, patients cannot be fully randomized as they are in RCTs. The proportion of patients with psoriasis was higher in the IL-17 inhibitor group, and although no statistically significant difference was observed, the proportion of patients with a history of uveitis or IBD was numerically higher in the TNF inhibitor group. These aspects reflect trends in biologic decisions in real-world clinical practice [33]. The diagnosis of AS is strictly regulated by the National Health Insurance Service in Korea, and only patients with AS who meet the modified New York criteria [13] are eligible for biologics insurance coverage. The male dominance and high HLA-B27 positivity in the baseline characteristics confirmed that the patient population in this study accurately reflected the typical characteristics of patients with AS. In Korea, the management of AS is based on both international and national guidelines [3, 33, 34]. As biologics are largely covered by insurance in Korea (90%), with comparable out-of-pocket costs across agents, differences in adherence rates attributable to cost are expected to be negligible. Considering the substantial coverage, the prescription of biologics may be primarily influenced by reimbursement criteria, which currently limit coverage to AS patients with a BASDAI score of ≥ 4. This study is noteworthy for providing findings that could be relevant to real clinical practice, as it analyzed data from real-world TNF or IL-17 inhibitor users among patients with AS over a relatively long median follow-up period of approximately 2.6 years. Further longitudinal studies with larger sample sizes are required to evaluate the long-term retention of biologics in patients with AS.

In conclusion, this study showed that the retention rates of TNF inhibitors tended to be higher than those of IL-17 inhibitors in AS patients with prior biologics experience. However, the number of previously exposed biologics and BASDAI emerged as significant factors influencing drug survival, rather than the class of biologics.

## Electronic supplementary material

Below is the link to the electronic supplementary material.

Supplementary Material 1

## Data Availability

No datasets were generated or analysed during the current study.

## Abbreviations

AS: Ankylosing Spondylitis
ASDAS: Ankylosing Spondylitis Disease Activity Score
BASDAI: Bath Ankylosing Spondylitis Disease Activity Index
BASFI: Bath Ankylosing Spondylitis Functional Index
BMI: Body Mass Index
CI: Confidence Interval
CRP: C-reactive Protein
ESR: Erythrocyte Sedimentation Rate
HR: Hazard Ratio
IBD: Inflammatory Bowel Disease
IL-17: Interleukin 17
NSAIDs: Non-Steroidal Anti-Inflammatory Drugs
SD: Standard Deviation
TNF: Tumor Necrosis Factor
