# Supplementary material for: Retention rate of TNF inhibitors versus IL-17 inhibitors in ankylosing spondylitis patients with prior biologics experience
Source: Arthritis Res Ther. 2025 Jul 4;27:135. doi: 10.1186/s13075-025-03601-z (PMC12232199; doi:10.1186/s13075-025-03601-z)
Supplement: Supplementary file 1 — Supplementary Material 1 [file 13075_2025_3601_MOESM1_ESM.docx]

| **Supplementary Table S1.** Factors associated with discontinuation of biologics in subgroup based on reasons for IL-17 inhibitor use | | | | | | | | |
| --- | --- | --- | --- | --- | --- | --- | --- | --- |
| Variables | Univariable | | | | Multivariable | | | |
|  | HR | 95% CI | | *P*-value | HR | 95% CI | | *P*-value |
| Age | 1 | 0.97 | 1.03 | 0.796 |  |  |  |  |
| Male gender | 0.42 | 0.19 | 0.92 | **0.030^*^** | 0.45 | 0.19 | 1.05 | **0.064** |
| BMI | 0.96 | 0.87 | 1.06 | 0.449 |  |  |  |  |
| HLA-B27 positivity | 0.37 | 0.13 | 1.10 | 0.074 |  |  |  |  |
| Disease duration | 1 | 1 | 1 | 0.751 |  |  |  |  |
| Peripheral arthritis | 0.71 | 0.31 | 1.61 | 0.408 |  |  |  |  |
| Number of previous biologics | 1.95 | 1.35 | 2.81 | **<0.001^**^** | 1.83 | 1.23 | 2.72 | **0.003^**^** |
| BASDAI | 1.36 | 1.03 | 1.80 | **0.033^*^** | 1.36 | 0.97 | 1.9 | **0.076** |
| IL-17 inibitor for reasons other than psoriasis aggravation  versus TNF inhibitor | 2.38 | 1.08 | 5.20 | **0.031^*^** |  |  |  |  |

^*^ *P* < 0.05, ^**^ *P* < 0.01.

IL-17: interleukin 17, HR: hazard ratio, CI: confidence interval, BMI: body mass index, HLA-B27: human leukocyte antigen B27, BASDAI: Bath Ankylosing Spondylitis Disease Activity Index, TNF: tumor necrosis factor.
